# Adaptive immunity, chronic inflammation and the clock

**DOI:** 10.1007/s00281-022-00919-7

**Published:** 2022-03-01

**Authors:** Kathryn J. Gray, Julie E. Gibbs

**Affiliations:** Centre for Biological Timing, Faculty of Biology Medicine and Health, University of Manchester, Manchester, M13 9PT UK

**Keywords:** Circadian, Adaptive immunity, Autoimmune disease, Lymphocyte

## Abstract

The adaptive arm of the immune system facilitates recognition of specific foreign pathogens and, via the action of T and B lymphocytes, induces a fine-tuned response to target the pathogen and develop immunological memory. The functionality of the adaptive immune system exhibits daily 24-h variation both in homeostatic processes (such as lymphocyte trafficking and development of T lymphocyte subsets) and in responses to challenge. Here, we discuss how the circadian clock exerts influence over the function of the adaptive immune system, considering the roles of cell intrinsic clockwork machinery and cell extrinsic rhythmic signals. Inappropriate or misguided actions of the adaptive immune system can lead to development of autoimmune diseases such as rheumatoid arthritis, ulcerative colitis and multiple sclerosis. Growing evidence indicates that disturbance of the circadian clock has negative impact on development and progression of these chronic inflammatory diseases and we examine current understanding of clock-immune interactions in the setting of these inflammatory conditions. A greater appreciation of circadian control of adaptive immunity will facilitate further understanding of mechanisms driving daily variation in disease states and drive improvements in the diagnosis and treatment of chronic inflammatory diseases.

## Introduction

The circadian clock is an internal timing mechanism which imprints daily rhythms on animal physiology. These oscillations in physiological processes such as sleep, feeding, metabolism and immunity allow animals to thrive in the 24-h environment generated by Earth rotating on its axis. Here, we review what is known about the rhythmic physiology of adaptive immune cells and discuss evidence for functional intrinsic clocks and rhythmic extrinsic signals directing this behaviour. We address bi-directional links between the clockwork machinery and the function of adaptive immune cells, both in health and disease. Recognising the daily rhythmicity of a number of chronic inflammatory diseases, we consider how circadian clocks contribute to these daily oscillations and ask how understanding circadian input into the adaptive immune system may benefit the diagnosis and treatment of inflammatory conditions.

### Adaptive immunity and the key cellular players

The immune system incorporates two functionally distinct, yet intertwining, arms of defence against pathogenic invasion or insult. The initial arm involves *innate defence* and includes maintaining barrier sites and non-specific killing of pathogens. Cells of the innate immune system recognise foreign bodies via their pathogen-associated molecular patterns (PAMPs), allowing an element of discrimination between self and non-self. Although crucial and effective, the first line of defence is not without limitations. The *adaptive defence* is far more fine-tuned, allowing specific recognition of foreign particles and the selective expansion of cells prepared to target specific pathogens and develop immunological memory. T and B lymphocytes are key component cells for adaptive immune responses with dendritic cells (DCs), which survey for foreign antigens, linking the innate and adaptive responses. Exogenous antigen is processed within DCs and displayed on their surface (via major histocompatibility complexes (MHC)). DCs then travel to lymph nodes where they interact with naïve T cells. Once in close proximity, the presented antigen joins with a corresponding T cell receptor (TCR) on the T cell surface; in the presence of a co-stimulatory signal, this triggers T cell activation and proliferation. If a B cell has encountered the same antigen, T cell-dependent B cell activation may occur (via CD40-CD40 ligand interaction) driving transformation of B cells to plasma cells, which secrete immunoglobulins.

### Adaptive immune cells and intrinsic clocks

Circadian rhythms are generated by the molecular clockwork machinery—a transcriptional translational feedback loop involving a small handful of genes and proteins (reviewed in [1]). In brief, the mammalian molecular clock involves two proteins CLOCK and BMAL1 which dimerise and bind to Ebox elements on the promoters of *Period (Per1-3)* and *Cryptochrome* (*Cry1* and *Cry2*) to enhance their transcription. The transcribed proteins PER1/2 and CRY1/2 dimerise and in turn act to inhibit the action of CLOCK/BMAL1 on their own promoters to limit transcription. This cycle takes approximately 24 h to complete. Additionally, CLOCK/BMAL1 enhance the transcription of two nuclear receptors called *Rev-erb* (α and β) and *Ror* (α, β and γ) which initiate (ROR) or inhibit (REV-ERB) the transcription of *Bmal1* respectively via a retinoic acid–related orphan receptor response element (RORE) within the promoter. The rhythmicity of the core clock is transferred onto physiological outputs via interaction with clock-controlled genes.

It has been well documented that cells of the innate immune system have this operational clockwork machinery which drives rhythms in cellular functions [2–4]. There is no doubt that cellular components of the adaptive immune system exhibit daily oscillations in behaviour, the most well-established example being 24-h rhythms in numbers of circulating lymphocytes [5, 6]. However, the evidence in favour or against functionally relevant clockwork machinery within adaptive immune cells is less abundant. Here, we discuss work in the field exploring whether adaptive immune cells possess functional intrinsic clocks.

### Clocks within T cells

At the highest level, T cells are divided by expression of surface proteins into CD4 + and CD8 + . CD8 + T cells are cytotoxic and can directly kill compromised cells and cancer cells. In contrast, CD4 + ‘helper’ T cells indirectly contribute to cellular destruction. Upon activation, CD4 + T cells differentiate into subsets, depending on the nature of the antigen and cytokines present within the local environment. Subsets identified to date include T helper1 (Th1), Th2, Th9, Th17, Th22, follicular helper T (Tfh) and regulatory T cells (Tregs). T helper subsets contribute to pro-inflammatory responses directed at eliminating specific microbial pathogens and activating other immune cell types. Tfh cells promote the survival and proliferation of germinal centre B cells. Tregs specialise in maintaining immune homeostasis and self-tolerance, dampening inflammation and preventing the development of autoimmune disease. Additionally, long-lived memory T cells can be derived from CD4 + and CD8 + T cells after antigen encounter. Work to date has demonstrated that some T cell subsets exhibit active rhythmic transcription of components of the core molecular clock. However, this has been limited to major T cell subsets, with scope remaining to further understand clock function within more specialised subsets.

#### CD4 + T cells

Definitive demonstration of a functional cell intrinsic clock within naive CD4 + T cells remains somewhat elusive. Bollinger and co-workers identified a trend for rhythmic clock gene expression in unstimulated cultured human peripheral CD4 + T cells sampled over a 24-h period, with *Rev-erbα*, *Per3* and *Bmal1* being the most rhythmic [7]. In contrast, Hemmers and Rudensky report only minor oscillations of the molecular circadian clock in murine CD4 + T cells, showing weak oscillations in *Bmal1* and *Rev-erbα* [8]. Notably, *Rev-erbα* rhythms persisted in the absence of BMAL1, suggesting the influence of extrinsic circadian factors driving these oscillations. One such factor might be endogenous glucocorticoids. Work by Torra and colleagues has demonstrated that within the liver, Rev-erb*α* transcript expression is under control of both the circadian clock and glucocorticoids [9]. Further work confirmed rhythmic expression of *Rev-erbα*, and also *Dbp* in murine CD4 + T cells [10]. Together, it seems any oscillations in the clockwork machinery in naïve CD4 + T cells are weak. It is possible that these are altered upon cellular activation. Indeed, 24-h rhythms in bioluminescence have been observed from CD4 + T cells isolated from spleen and thymus of PER2::luciferase mice stimulated with phorbol 12-myristate 13-acetate (PMA) [7]. Furthermore, it has been shown that anti-CD3e/anti-CD28 stimulation can induce PER2 bioluminescence, indicating direct coupling of core clock genes to extrinsic T cell stimulation [11]. Despite lack of conclusive evidence of a functional clock within naïve CD4 + T cells, it is well recognised that genetic deletion of *Bmal1* in CD4 + cells dampens the rhythmic function of these cells and impacts on temporal gating of adaptive immune function [10] (see ‘Lymphocyte trafficking’ section).

#### CD8 + T cells

Nobis et al. reported intrinsic rhythms in naive murine CD8 + T cells [12]. Purified CD8 + cells from PER2::luciferase mice exhibited rhythms in bioluminescence. Furthermore, RNA sequencing revealed robust oscillations in some components of the molecular clock (*Per2*, *Rev-erbα* and *Dbp*) in CD8 + cells; however, other clock genes (e.g. *Cry1/2* and *Bmal1*) could not be classified as rhythmically expressed.

#### Tregs

Naïve murine Tregs do not appear to possess a functional intrinsic clock [11]. Although, mirroring observations in CD4 + T cells, *Rev-erbα* did show daily rhythms in transcript expression, but no other core clock gene examined behaved rhythmically.

### Clocks within B cells

Studies examining cellular clocks within B cells are lacking in number. Hemmers and Rudensky observed minor fluctuations in *Per1* reporter (*Per1*^venus^) expression over 24 h in murine B cells, but did not detect any abnormality in B cells lacking BMAL, leading them to conclude that functional clockwork machinery, if present, is not essential for normal B cell development [8].

### Clocks within DCs

DCs sit at the interface between the innate and adaptive immune system and as antigen presenting cells (APCs) prime the adaptive immune response. Early work in the field established oscillations in core clock genes within splenic DCs harvested from mice over a 24-h cycle [13]. More recently, Hopwood et al. demonstrated robust rhythmic PER2::luciferase bioluminescence from murine cultured bone marrow–derived DCs [14], confirming intrinsic rhythmicity.

### Circadian regulation of adaptive immunity

Despite limited evidence for multiple cellular components of the adaptive immune system possessing functional cell intrinsic clocks, this defence system exhibits daily variation in cellular composition, cell trafficking and responses to insult. Here, we explore our current understanding of rhythmicity within the adaptive immune system, considering circadian control of homeostatic processes (such as maintenance of immune cell populations) and responses to challenge.

### Th17 development

The circadian clock is important for the lineage specification and maintenance of intestinal Th17 cells. These cells make an important contribution to defence against extracellular bacteria, but also play a key role in the development of autoimmune diseases. Lineage specificity of Th17 cells is regulated by the transcription factor RORγt, which is a short isoform of RORγ. Expression of RORγt is limited to lymphoid cells and neutrophils (in contrast RORγ is not expressed in haematopoietic cells, but found more widely in peripheral tissues) [15]. Whilst expression of *Rorγ* is regulated by CLOCK/BMAL, this heterodimer cannot activate transcription of the RORγt promoter [16]. However, within activated murine CD4 + T cells, *Ror*γt expression exhibits diurnal variation [17], and this is reported to be due to diurnal variation in binding of nuclear factor interleukin 3 regulated (NFIL3) to the promoter (see below).

Studies in mice have demonstrated that REV-ERBα negatively regulates Th17 cell development both directly and indirectly. REV-ERBα can itself compete with RORγt to modulate Th17 signature genes [18], but also regulates expression of NFIL3, a transcription factor which directly binds and represses the RORγt promoter [17]. A consequence of these interactions is diurnal variation in numbers of Th17 cells within the lamina propria. Yu and colleagues demonstrated higher frequencies of intestinal Th17 in the day versus night [17]. Additional circadian mechanisms have been recognised to regulate enteric Th17 cell numbers through regulation of cell trafficking. Th17-specific IL17A secretion induces CCL20 production from the small intestine, which facilitate Th17 migration back to this site [19]. CCL20 expression exhibits circadian rhythms and likely contributes to diurnal variation in Th17 cell frequency within the small intestine [20].

### Lymphocyte trafficking

T and B cells traffic through the lymph nodes (LN) in a circadian manner. In mice, lymphocyte accumulation in LNs peaks at the onset of night (Zeitgeber Time (ZT)12–13) and egress occurs during the day [10, 21]. Multiple mechanisms have been shown to facilitate this daily rhythm, with stress-associated hormones being at the forefront. The first is via adrenergic control [22]. Neural inputs to lymphocyte β_2_-adrenergic receptors inhibit their egress from LNs at night, promoting retention (discussed in more depth below). In addition, daily rhythms in circulating glucocorticoids contribute to diurnal oscillations in T cell distribution [21, 23]. Activation of the glucocorticoid receptor (GR) induces IL-7R expression on T cells and upregulates expression of the chemokine receptor CXCR4 which binds CXCL12 and promotes homing to lymph nodes [21].

In addition to these rhythmic cell extrinsic signals, there is clear evidence for a role for cell intrinsic clocks directing rhythmic lymphocyte trafficking. Druzd and colleagues demonstrated diurnal variation in expression of two trafficking receptors on lymphocytes—CCR7 and sphingosine-1-phosphate receptor 1 (S1P1) [10]. CCR7 binds CCL21 to promote lymphocyte homing to lymph nodes. In contrast, S1P1 binds sphingosine-1-phosphate, which guides lymphocytes out of LNs and into efferent lymphatics. Critically, cell-specific ablation of *Bmal1* in T cells results in loss of diurnal rhythms in expression of CCR7 and *S1pr1* (which encodes S1P1) and loss of rhythms of lymphocyte trafficking [10]. Together, this suggests concerted action between cell intrinsic and extrinsic circadian signalling driving rhythmicity in a homeostatic process which is critical for immune surveillance and responses to antigen presentation. Of course, the role of extrinsic rhythmic signalling extends beyond the direct influence on chemokine signalling pathways and trafficking receptors to include influence on the core clock itself (reviewed in [24]). One example being the influence of glucocorticoids on *Per1/2* expression and *Rev-erbα* [9, 25–27], which may then influence clock dependent trafficking mechanisms.

A second important aspect of lymphocyte trafficking which likely contributes to the daily variation in circulating numbers is movement between the bone marrow (BM) and blood. The BM contains mature T cells (CD4 + CD8 + and Tregs) and these make up approximately 3–8% of total nucleated BM cells [28]. Trafficking between compartments is well established [29] but the influence of the circadian clock is not yet fully understood. Given the influence of stress mediators (catecholamines and glucocorticoids) on trafficking of haematopoietic stem cells between BM and blood (reviewed in [30]), it seems likely that these rhythmic signals may also influence T cell trafficking from bone marrow. Indeed, work across humans and mouse models demonstrates a role for the glucocorticoid-regulated chemokine receptor CXCR4 [21] in retention of Tregs within the bone marrow (which expresses the ligand CXCL12) [31].

### Response to vaccination

Vaccinations utilise the adaptive immune system to generate antibodies towards a target antigen [32]. Broadly, a small amount of the foreign antigen is introduced to the body, provoking APCs to traffic to lymph nodes, where they present the antigen to T cells. Activated T cells drive the development of B cells and concurrently, B cells are stimulated (via the B cell receptor) by soluble antigen. B cells provide an antibody response. Memory B cells are generated, which mediate long-term immune memory, along with CD8 + memory T cells which are primed to proliferate rapidly when they next encounter a pathogen. Evidence is emerging from clinical studies highlighting that time of day influences vaccine responses. Studies suggest that subjects exhibit enhanced immunogenicity after influenza, hepatitis A and bacillus Calmette-Guerin (BCG) vaccination in the morning compared to the evening [33–35]. Studies in animal models (utilising immunisation with an autoantigen) similarly show that responses by the adaptive immune system to immunisation follow a circadian rhythm [10, 12, 36]. In part, this is due to oscillations in numbers of CD4 + T cells in lymph nodes at the time the animal encounters an antigen, which is influenced by multiple rhythmic signals (as discussed above). Work by two independent groups has determined that in a model of experimental autoimmune encephalitis (EAE; whereby mice are immunised with a MOG_35-55_ peptide in an adjuvant to induce an autoimmune response resulting in inflammation of the CNS), reactions to this immunisation are heightened if the antigen is encountered during the day (ZT6-ZT8) versus the night. This timing is perhaps counterintuitive in light of data from human studies, when encountering antigen during the early active phase provokes a greater response.

The circadian clock intrinsic to CD8 + T cells plays a role in regulating the magnitude of their response to antigen presentation [12]. Using a model whereby antigen is presented to T cells by bone marrow–derived DCs loaded with OVA_257-264_ peptide, Nobis and colleagues show that CD8 + T cell expansion in responses to vaccination is heightened at circadian time (CT)6 (mid-rest phase) compared to CT18 (mid-active phase). Further studies whereby vaccinated mice were challenged with a lethal dose of OVA-expressing *Listeria monocytogenes* showed enhanced protection after vaccination at CT6. CD8 + T cell-specific deletion of *Bmal1* abolishes circadian variation in CD8 + T cell expansion. Transcriptomics revealed circadian regulation of the TCR-dependent signalling pathway, thereby imparting temporal gating onto cell activation, proliferation and acquisition of effector function.

### Response to parasite infection

**Fig. 1:**
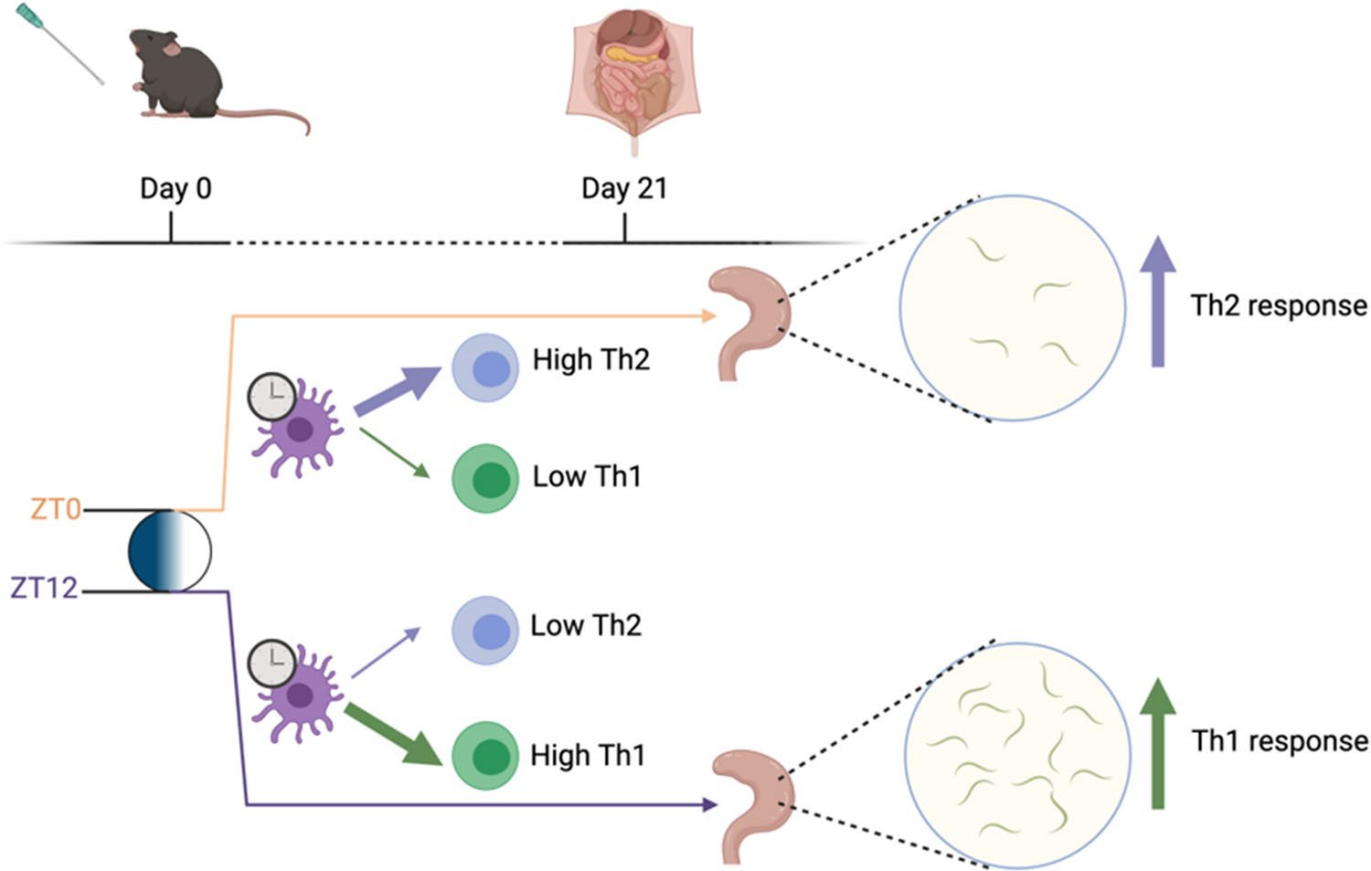
*T. muris* infection outcome is determined by infection time of day and the DC clock. When mice are infected with *T. muris* at ZT0, their DC clocks direct a Th2 response, leading to increased worm clearance measured by low caecal worm burden 21 days after infection. When mice are infected with *T. muris* at ZT12, their DC clocks direct a Th1 response, leading to decreased worm clearance measured by high caecal worm burden 21 days after infection

Murine responses to parasitic infection vary dependent on time of encounter (Fig. 1). This has been demonstrated using an infection model with *Trichuris muris* (a ceacal dwelling parasite) [14]. Here, DC presentation of antigen stimulates T cells to polarise towards a Th1 or Th2 response (dependent on factors including cytokine signal and antigen dose) with a Th2 response required for efficient worm expulsion. Hopwood et al. demonstrated a difference in worm expulsion rates 21 days post infection dependent on time of infection. Those animals infected at ZT0 (lights on) showed a strong Th2 bias and faster worm expulsion than those infected at ZT12 (lights off). In mice lacking *Bmal1* specifically in CD11c + cells (a cell subset that is largely composed of DCs), this difference in worm burden was lost, favouring development of a protective Th2 response across the day. RNA sequencing showed down-regulation of IL12 pathways as a consequence of *Bmal1* deletion in DCs. IL12 is critical for promoting Th1 polarisation, which could help explain the altered parasite expulsion ability. The study demonstrates clock influence over adaptive immune responses to parasite infection, and highlights the importance of clocks within DCs for tuning downstream T cell responses.

### Circadian disruption causes changes to the adaptive immune system and is associated with chronic inflammatory disease

Circadian disruption can be brought about experimentally through genetic targeting or environmental manipulation. Genetic targeting takes the form of deletion or mutation of one of the core clock genes, often *Bmal1* deletion or *Clock* mutation. Global targeting of these genes leads to varying effects on rhythms in rest-activity cycles but often impacts on the adaptive immune system. These effects on the immune system can be a consequence of direct action of that clock gene on immune processes integral to maintain homeostasis. An example here is the influence of *Rev-erb* α and β on DC development and maturation [37]. Deletion of *Rev-erb α/β* enhances expression of maturation markers and pro-inflammatory cytokines even under naïve conditions. Multiple studies have explored consequences of circadian disruption on development of chronic inflammatory diseases both in mouse and humans, and these are discussed below.

### Effects of circadian disruption on development of chronic inflammatory disease

Many chronic inflammatory diseases are sensitive to perturbations in circadian rhythms, and recent advances are beginning to unravel how the clockwork machinery within individual cell types is important for balancing inflammatory responses and restraining inflammation. Of note, there is a growing field of literature investigating the role of the clock in the development and pathogenesis of inflammatory bowel disease, inflammatory arthritis, asthma and multiple sclerosis.

#### Inflammatory bowel disease (IBD)

IBD encompasses ulcerative colitis (UC) and Crohn’s disease (CD). UC is a chronic autoinflammatory condition of the colon and, although causes remain ultimately unknown, it has been suggested that UC may be a consequence of excessive inflammation initiated by a bacterial or viral infection. CD also involves chronic autoinflammation of the intestinal tract but is not limited to the colon and instead can affect anywhere from the mouth to the anus. Causes of CD are also unknown but likely multifaceted and potentially include hereditary factors.

There is a paucity of human data directly linking circadian disturbance with increased incidence or severity of IBD. However, assessment of IBD subjects through questionnaires around sleep and chronotype revealed an association of IBD-specific complications and/or lower quality of life with later chronotype, social jet lag, sleep debt and inconsistent meal timings [38]. There is also some evidence linking the core molecular clock to development of IBD. Mazzoccoli and colleagues found a possible link between a PER3 polymorphism and susceptibility to both UC and CD and also with phenotypic characteristics of CD such as higher use of immunosuppressives [39]. Animal models, such as dextran sodium sulphate (DSS)–induced colitis, have provided evidence that environmental manipulation driving circadian disruption can enhance disease severity in this pre-clinical model of colitis. Two studies [40, 41] demonstrate the negative impact of circadian perturbation (in the form of prolonged periods of phase shifts) on colitis severity.

**Fig. 2:**
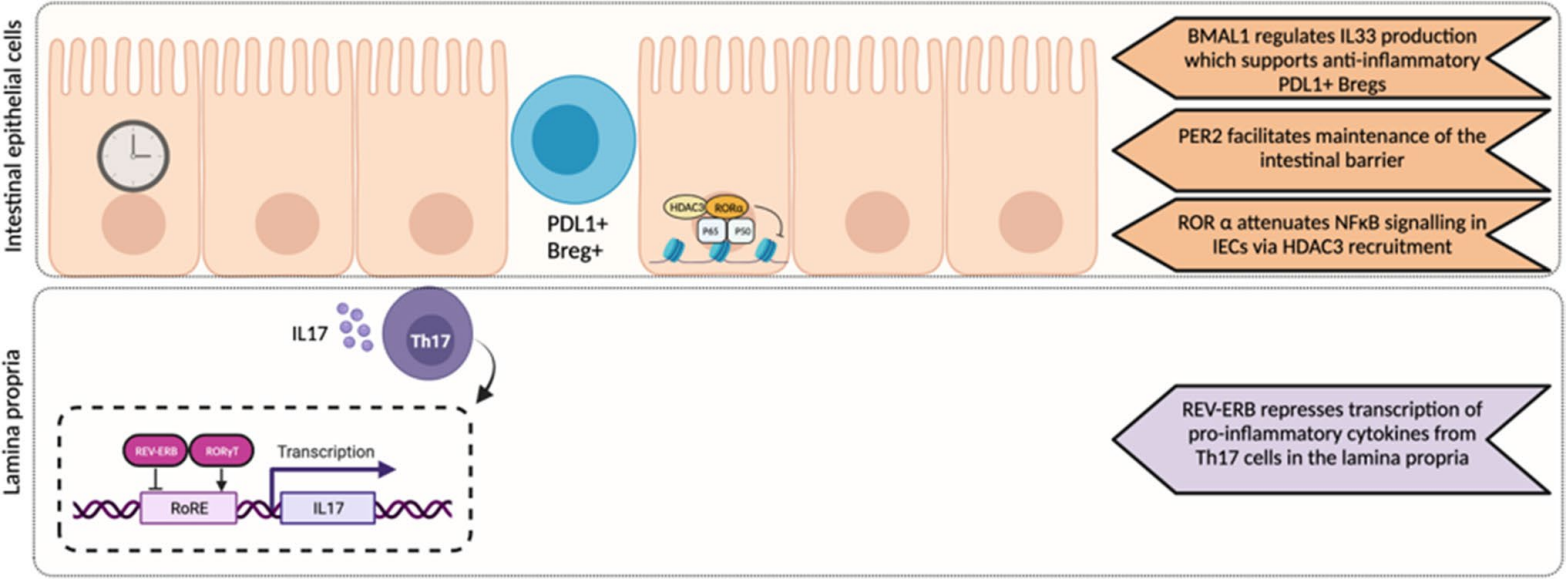
The circadian clock regulates multiple mechanisms contributing to gastrointestinal inflammatory responses in experimental models of colitis. PER2 is important for maintenance of intestinal epithelial cells (IECs) which form a barrier between the gut microbiome and the host. Within the IECs RORα attenuates inflammatory responses via action on NFκB signalling. Intraepithelial regulatory B cells (Bregs) act to repress local inflammation, and maintenance of this cell population is regulated by BMAL-driven IL33 production. Finally within the lamina propria, Th17 cells drive local inflammatory responses in colitis, which is negatively regulated by REV-ERBα

Multiple nodal points between inflammatory processes underlying IBD and the clock have been identified and include PER1/2, BMAL1, REV-ERBα and RORα (Fig. 2). PER1/2^−/−^ mice showed an aggravated course of colitis upon DSS challenge. This is considered to be a consequence of PER1/2 playing a prominent role in maintenance of the intestinal barrier, and in particular the maintenance of secretory intestinal epithelial cells (IECs) which are key players in the pathology of IBD [42]. Other studies have highlighted the importance of the clock within IECs for restraining responses to inflammation. Mice lacking RORα specifically in IECs demonstrate excessive inflammation and tissue damage in response to DSS. This is a consequence of RORα acting as a transcriptional repressor of inflammatory genes by attenuating NF-κB signalling [43]. Mice lacking BMAL1 globally present an enhanced response to DSS-induced colitis [41, 44]. Liu et al. highlighted that BMAL1 regulates rhythmic production of IL33 within the intestinal microenvironment which supports programmed cell death 1 ligand (PDL1) + Breg + cells within the intestinal intraepithelial lymphocytes which act to inhibit excessive inflammation. Global REV-ERBα^−/−^ mice exhibit more severe DSS-induced colitis and this is in part a consequence of the influence of this nuclear receptor on the NOD-like receptor family pyrin domain containing 3 (NLRP3) inflammasome. REV-ERBα represses *Nlrp3* transcription (a component of this protein complex) via a REV-ERB response element (RevRE) and two NFκB binding sites in the promoter. Additionally, REV-ERBα represses transcription of p65 (a subunit of NFκB) [41]. Furthermore, as discussed above, REV-ERBα negatively regulates Th17 development and IL17 production (thus having a protective effect in experimental models of colitis) [18].

Together, it is clear that the circadian clock within the gut plays a critical role in maintenance of a number of key cell types critical for the regulation of barrier function, not just adaptive immune cells but also tissue-resident IECs. Circadian disruption is likely to impact on these processes and thus drive increased susceptibility to chronic inflammatory disease or an enhanced response to established disease.

#### Rheumatoid arthritis (RA)

RA is an autoimmune disease causing chronic localised inflammation within the joints, resulting in joint pain and stiffness with eventual tissue remodeling if left untreated. There is some evidence to link circadian disruption induced by shift work with the development of RA [45, 46]. Additionally, a correlational study identified RA patients as having a slightly earlier chronotype, with mid-point of sleep on free days occurred 23 min earlier than subjects from the general population [47]. Whilst this further supports a link between the clock and RA, as the authors point out, this could be a consequence of earlier sleep onset and/or earlier sleep end, and the consequences of early morning joint pain on sleep end should be considered here. Analysis of circadian rhythmic hormones (melatonin and cortisol) has also been used to assess circadian disruption in RA. Circulating nighttime melatonin levels (secreted by the pineal gland at night under control of the SCN) peak 2 h earlier in RA patients compared to healthy controls [48]. Whilst onset of melatonin secretion under dim light (dim light melatonin onset, DLMO) is an accurate marker for assessing circadian phase [49], it is not clear whether in this study volunteers were exposed only to dim light prior to sampling as recommended. Analysis of cortisol rhythms in RA patients has shown inconsistent results. Cortisol is a glucocorticoid produced by the adrenals under control of the hypothalamic pituitary adrenal (HPA) axis (the rodent equivalent being corticosterone). Cortisol/corticosterone release is under control of the central clock and circulating levels exhibit 24-h variation. Crofford and colleagues report normal circadian rhythms of cortisol [50] in RA patients, whilst Neeck et al. observed phase advances in patients with low to medium disease activity and more disrupted rhythms in patients with high disease activity [51]. General consensus is that the HPA axis remains rhythmic in RA but is unable to mount an appropriately enhanced response to combat joint inflammation [52, 53].

Data from animal studies has linked specific elements of the circadian clock to the pathology of RA. CRY has been shown to repress joint inflammation in a mouse model of collagen antibody–induced arthritis (CAIA). CRY1/2^−/−^ mice exhibit aggravated disease which is linked to increased TNFα production from splenic lymphocytes leading to increased activation of CD3^+^ CD69^+^ T cells [54]. Further studies have shown that joint fibroblast-like synoviocytes (FLS) from CRY1/2^−/−^ animals exhibit enhanced inflammatory responses and targeted knockdown of CRY1/2 in human FLS has the same effect [55]. Loss of BMAL in FLS also has a negative impact on inflammatory arthritis, with targeted mice exhibiting enhanced pro-inflammatory responses and increased recruitment of monocytes to inflamed joints [56]. Although the clock plays a role in regulating processes underlying RA, the involvement of clocks within tissue-resident and innate immune cells appears more prominent than timing mechanisms within adaptive immune cells.

#### Asthma

Asthma is a pulmonary condition in which inflammation-driven airway narrowing affects the ability to breathe. Disturbances to the clock affects development of disease, with a recent study from Maidstone and colleagues, utilizing data from the UK Biobank, linking night shift work with an increased risk of asthma [57]. Furthermore, a recent study has identified 3 polymorphisms in TIMELESS and two haplotypes which associate with asthma risk in childhood [58]. Together, it is clear that environmental and genetic manipulations which lead to circadian disturbances are risk factors for the development of asthma. In support, work by Ehlers utilising a mouse model of Sendai virus (SeV) to model chronic airway changes reminiscent of asthma showed that a chronic jet lag protocol, which disrupts temporal clock gene expression within the lungs, results in increased airway resistance and methacholine sensitivity in the latter stages of the model [59].

BMAL1 within myeloid cells negatively regulates allergic lung inflammation in a model of ovalbumin-induced allergic asthma [60]. LysM-Bmal1^−/−^ mice respond to the challenge with increased numbers of eosinophils and IL-5 expression (a key mediator of eosinophil activation). Further work revealed that macrophages from these animals exhibit elevated responses to both typical M1 challenge (lipopolysaccharide) and M2 challenge (IL4) suggesting that BMAL1 in macrophages is important for mediating macrophage-eosinophil crosstalk. Notably, this study highlights a role for cell intrinsic clocks within innate (rather than adaptive) immune cells for driving rhythms in allergic inflammation.

#### Multiple sclerosis (MS)

MS is an autoimmune disease of the CNS, whereby activated T cells drive demyelination of the neurons of the brain and spinal cord. As with the autoimmune diseases discussed above, there is evidence to link development of MS to disruptions of the circadian clock. Firstly, a Danish study in a teenage population (15–19 years) associates the development of MS to shiftwork [61]. Secondly, polymorphisms in core clock genes (*Bmal1* and *Clock*) are more common in MS patients than healthy controls [62].

Additionally, the circadian hormone melatonin, which shows altered circadian profiles dependent on latitude and season, has been implicated in contributing to the pathogenesis of MS. Whilst there is no evidence for disturbances in circadian rhythms in melatonin in MS patients, lower melatonin levels have been associated in exacerbations in relapsing–remitting patients [63]. Studies in animals around the role of melatonin in the pathogenesis of MS offer conflicting conclusions, with some support of an anti-inflammatory effect, but this may be age-dependent [64]. Melatonin administration in the murine EAE model protects against disease development by altering peripheral and central T effector and Treg responses [65]. In humans, there is a report of melatonin supplements improving disability status in a primary progressive MS patient [66], but further clinical trials are lacking.

There is a noted increase in prevalence of MS in countries of high latitude, where there are extended periods of short days and long nights [67]. There is general consensus that this latitudinal gradient of MS prevalence is linked to sun exposure. It has been postulated that this effect of sunlight may be mediated by melatonin [68], whereby long nights induced prolonged melatonin secretion. Given the potential beneficial influence of melatonin on MS, it would seem that this is an unlikely mediator of the latitudinal gradient effect. A more likely and well-supported mediator is vitamin D (which requires UVB radiation for synthesis) [69].

Studies utilising EAE as a murine model of MS further highlight links between the molecular clock and disease pathology. Global REV-ERBα^−/−^ mice exhibit an earlier onset and exacerbated disease in EAE [18]. Loss of REV-ERBα results in elevated RORγt^+^ cell frequency in the CNS, and reduced anti-inflammatory Tregs. Conversely, deficiency in both RORα and RORγ protects against EAE as a consequence of impaired development Th17 cells [70]. Together this highlights a role for the circadian clock in regulating Th17/Treg balance and maintenance of immune tolerance. As discussed above, studies have shown that time of disease induction in EAE affects outcomes, with increased disease severity after immunisation during the light phase [10, 36]. Cell-specific ablation of *Bmal1* reveals roles for cell intrinsic clockwork machinery within T cells [10] and myeloid cells [36] for this temporal gating.

### Chronic inflammation affects the molecular clock

Common across these chronic inflammatory diseases is that the core clockwork machinery becomes dampened at the site of inflammation. For example, studies in human tissue have shown perturbations of clock genes and proteins within the synovial membrane [71] and specifically FLS [72] in RA patients. A mouse model of inflammatory arthritis additional shows clear evidence of down-regulation of core clock genes in inflammatory cells recruited to the joints, including macrophages and neutrophils [11]. Similarly, there is evidence for dampened clock gene expression within both the site of inflammation [41, 73, 74] and within the periphery [74, 75] in colitis and within the spinal cord in EAE [36]. In asthma patients, there is evidence for altered clock gene expression patterns in the respiratory system. Ehlers and colleagues report a decreased expression of 6 core clock genes (but increased *Clock* expression) in bronchial brushings from adult asthma patients compared to time matched controls [59]. A study of peripheral blood mononuclear cells in patients with bronchial asthma revealed down-regulation of eight circadian clock genes compared to healthy individuals [76]. Again, suggesting any effects on the molecular clock may extend from the site of local inflammation into the periphery.

It is hard to establish whether altered clock gene expression is cause or consequence of chronic inflammation, but it is clear that acute inflammation impacts on the clock machinery both at a transcriptional and post-transcriptional level [11, 77]. The transcription factor NFκB has been highlighted as a key mediator of inflammation-induced circadian disruption [78]. Activation of NFκB disrupts circadian transcription via inhibition of the negative arm of the clock (*Per*, *Cry* and *Rev-erb*). ChIP sequencing of p65 in the mouse liver after lipopolysaccharide stimulation revealed co-localisation of NFκB to sites occupied by CLOCK/BMAL, leading to circadian disruption [78]. Further work in human omental adipocytes has shown binding of p65 to the PER2 promoter, which prevent BMAL1 binding, thus inhibiting *Per2* transcription and impairing circadian clock function [79]. In addition, clock proteins are sensitive to inflammation, with loss of REV-ERBα protein in lung tissue 2- to 4-h post-lipopolysaccharide stimulation [77]. This rapid degradation of REV-ERBα is promoted by the 26S proteasome. It remains to be seen whether these mechanisms underlie clock disruption in the setting of chronic inflammatory disease. Given the importance of the clock in regulating immune cell function, disruption is likely to have downstream impact on disease progression and potentially contribute to development of co-morbidities.

### Rhythms in chronic inflammatory disease

There is mounting evidence that circadian disruption is associated with chronic inflammatory disease and in the setting of chronic inflammation, cell intrinsic clocks become dampened. Yet, perhaps paradoxically, these diseases often present as circadian rhythmic, with daily oscillations in disease symptoms and markers. The best examples here are asthma and RA.

#### Asthma

The symptoms of asthma are well recognised as exhibiting diurnal variation. Pulmonary function (e.g. peak expiratory flow rate and forced expiratory volume in 1 s (FEV1)) in healthy individuals is lowest during the night, and this diurnal variation is exaggerated in asthma patients [80–83]. Experimental studies in human volunteers maintained under carefully controlled laboratory conditions (thus eliminating environmental factors and behaviours that normally cycle in tandem with the circadian system) confirm a role for the circadian clock in driving nocturnal worsening of asthma [84]. Volunteers maintained in dim light in either a 38-h constant routine (continuous wakefulness with fixed isocaloric snacks across time) or a 196-h forced desynchrony protocol (where sleep occurs across all circadian phases over the study days) still exhibited circadian peaks in pulmonary function, with reduced function during the biological night. Recent work has begun to explore mechanisms by which the pulmonary clock drives daily variation in asthma symptoms. Durrington and co-workers describe how daily variation in airway hyperresponsiveness (defined as increased sensitivity and reactivity of the airways to stimulation) in response to allergic challenge is mediated by REV-ERBα in mice [85]. This nuclear receptor has demonstrated action on expression of muscarinic receptors which contribute to the control of smooth muscle tone in the lung. Thus, circadian variation in airway hyperresponsiveness may be driven via circadian regulation of the cholinergic system. There is also circadian variation in the inflammatory milieu within the asthmatic lung. Clinical studies have shown increased numbers of CD4 + T cells in bronchoalveolar samples collected in the early morning (04:00) compared to afternoon (16:00) in mild asthma [86] and increased numbers of eosinophils in the sputum in the morning [83, 87]. Mechanisms driving this rhythmic inflammatory response are yet to be elucidated. One might postulate the involvement of rhythmic endogenous glucocorticoids. However, early studies in humans suggest that preventing the night time fall in corticosteroids via cortisol infusion does not prevent nocturnal asthma symptoms [88].

#### Rheumatoid arthritis

**Fig. 3:**
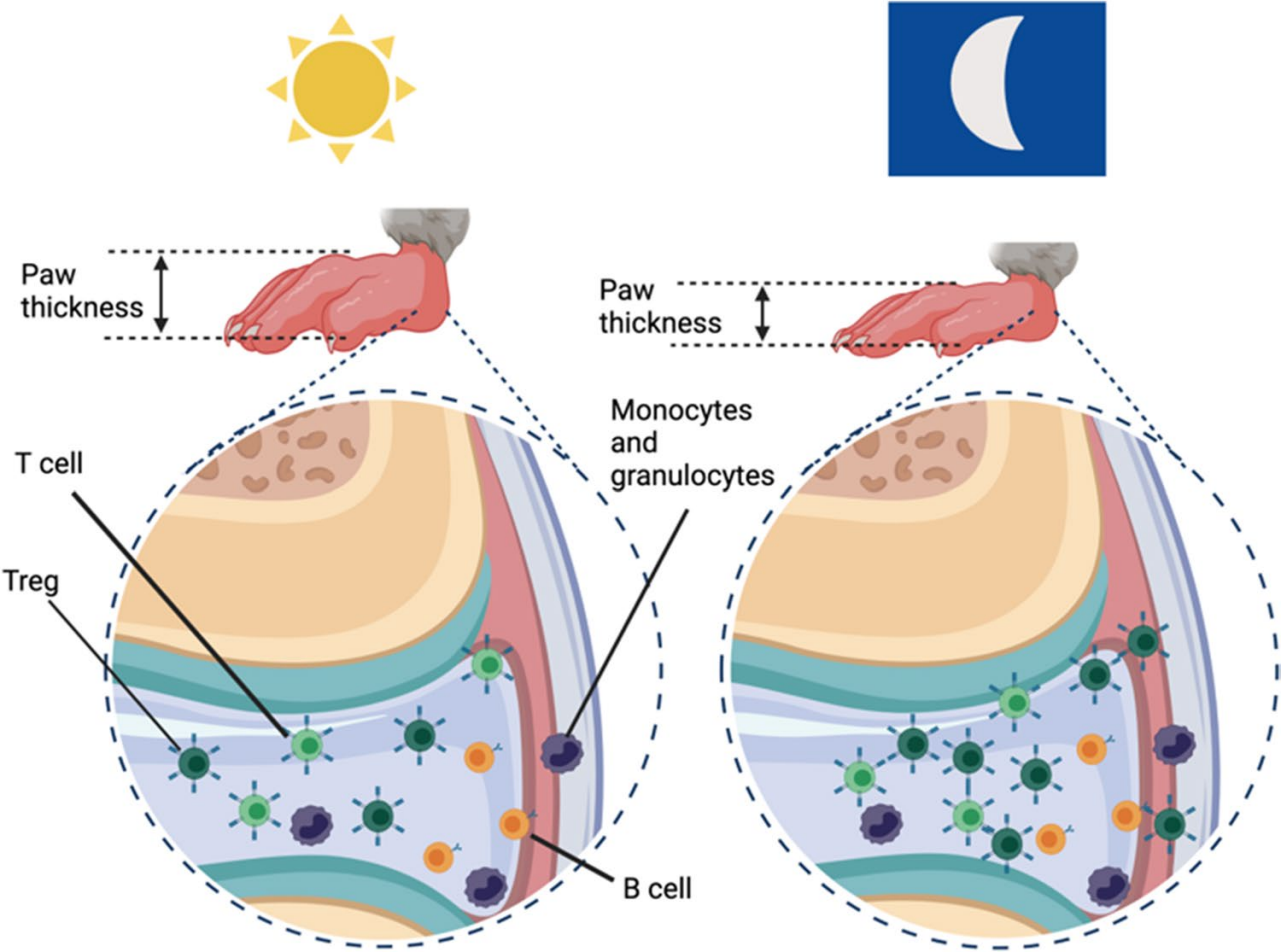
Regulatory T cells confer a circadian signature onto murine inflammatory arthritis. During the rest phase, arthritic mice present with an enhanced disease state, including increased paw thickness. During the active phase, paw thickness is decreased, and there is an increase in numbers of anti-inflammatory regulatory T cells. There is no diurnal variation in numbers of other lymphocytes

RA patients show diurnal variation in disease symptoms and biomarkers, with joint pain and stiffness heightened in the morning [89]. This diurnal variation correlates with daily oscillations in circulating pro-inflammatory cytokines such as interleukin 6 (IL6) [90, 91]. Additionally, analysis of peripheral blood cell populations in female RA patients reveals alteration in rhythms in lymphocyte frequency, with both loss and gain of rhythmicity of discrete subsets [92]. For example, rhythms in numbers of circulating effector CD4 + and CD8 + T cells are lost, but emerge in numbers of circulating B cells (CD20 + CD27 + memory and CD20 + HLA-DR + activated B cells). These data extend our understanding of the rhythmic nature of the disease beyond symptoms, to encompasses circulating humoral factors, cytokines and cells. Studies have shown that a pre-clinical mouse model of inflammatory arthritis (collagen induced arthritis, CIA) exhibits the same phenomenon, with levels of multiple circulating cytokines peaking during the day (rest phase for mice) and receding at night [55]. Additionally, transcription of pro-inflammatory cytokines within inflamed joints is rhythmic, mirroring observations in the periphery and highlighting local rhythmicity within inflamed tissue. Further work highlights a key role for Tregs for driving rhythmic inflammation within the joints [11] (Fig. 3). Tregs accumulate within murine inflamed joints during the dark phase, where they actively repress inflammation [11]. As discussed above, naive Tregs are not inherently rhythmic and do not appear to possess their own time-keeping machinery. Yet, these cells are demonstrably circadian regulated suggestive that these rhythms may be driven by rhythmic extrinsic factors.

### Rhythmic extrinsic signals may drive rhythms in chronic inflammatory disease

**Fig. 4:**
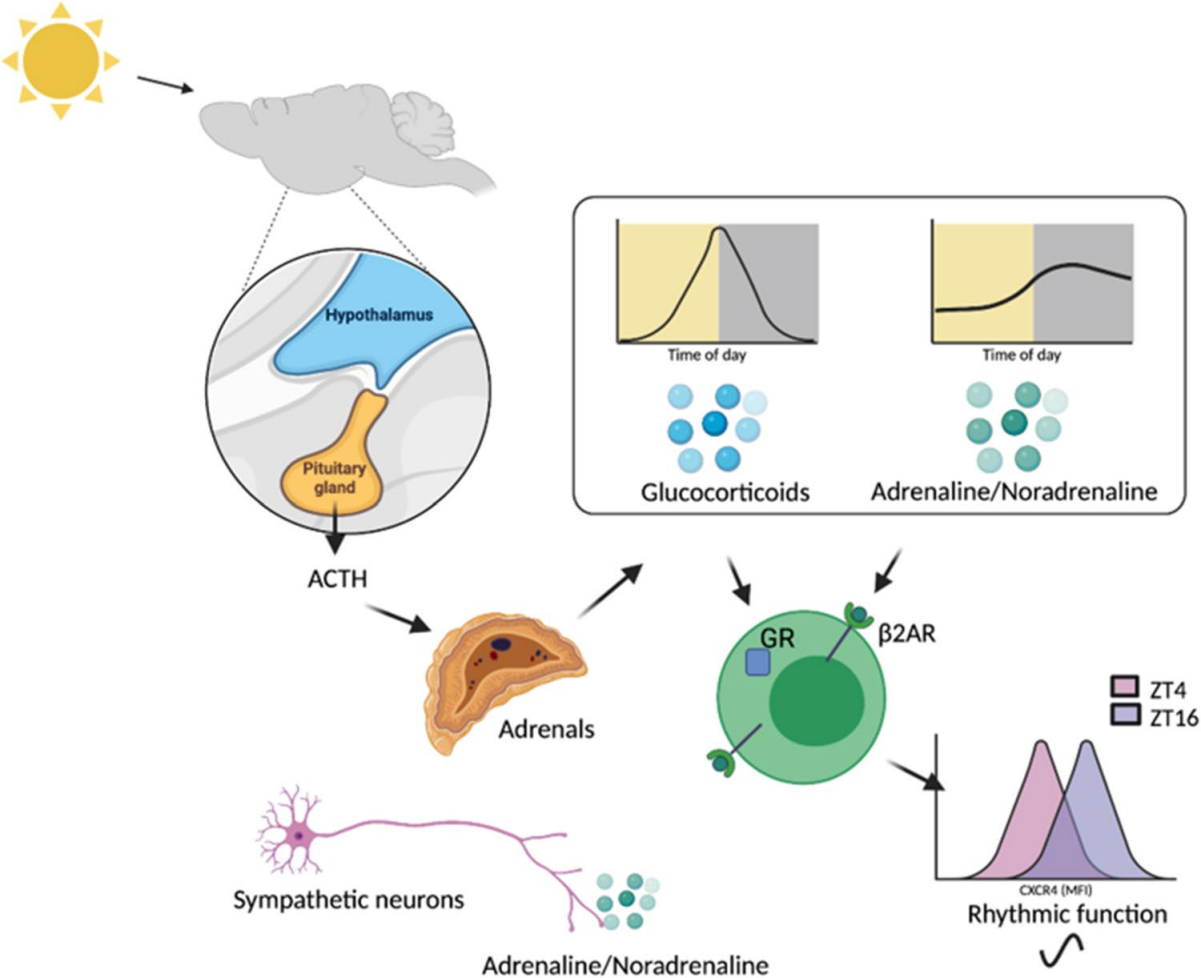
Cell extrinsic rhythmic signals drive daily variation in immune cells. Glucocorticoids are produced in a rhythmic manner via action of the SCN on the hypothalamic pituitary adrenal (HPA) axis. Catecholamines are produced by the adrenal and the sympathetic nervous system and circulating levels also exhibit diurnal variation. Both glucocorticoids and catecholamines influence the function of the adaptive immune system. For example diurnal variation in glucocorticoid levels drive rhythms in CXCR4 expression on the surface of T cells and B cells. Furthermore, activation of β2AR on lymphocytes enhances the responsiveness of CXCR4; thus, daily catecholamine rhythms contribute to diurnal variation in lymphocyte function. Adrenocorticotropic hormone (ACTH); glucocorticoid receptor (GR)

There is clear evidence that clockwork machinery within key effector cells, resident at the site of inflammation, plays an important role in repressing inflammation in the setting of chronic inflammatory disease. However often these are not adaptive immune cells themselves, but specialised tissue-resident cells such as IECs or FLS. However, we must also consider the importance of cell extrinsic rhythmic signals on the function of immune cells in the context of adaptive immunity and chronic inflammation. These rhythmic, non-photic time-cues (or ‘*zeitgebers*’) may take many forms including steroid hormones and adrenergic signals (Fig. 4).

#### Steroid hormones

Glucocorticoid production is under control of the circadian clock. These steroid hormones are secreted in a pulsatile fashion by the adrenal gland with levels peaking at the onset of activity (early morning in diurnal animals and early night in nocturnal rodents). Diurnal rhythms are maintained through SCN input to the HPA axis. Glucocorticoids have well-defined immunosuppressive activity on the adaptive immune system, not only altering effector function of T cells but also regulating T cell responsiveness by attenuating T cell receptor signalling [93]. Conversely, glucocorticoids also demonstrate immune-enhancing effects on the adaptive immune system [94]. Examples here include enhancing expression of the IL-7 receptor α chain (IL-7α) on T cells to promote survival via binding to glucocorticoid response elements (GRE) within the non-coding conserved sequence 1 (CNS-1) on the promoter [95–97]. Furthermore, studies in mice and humans have revealed that glucocorticoids also support immune homeostasis by inducing expression of the chemokine receptor CXCR4, which regulates T cell migration [98] and exchange of mature B cells between blood and bone marrow [99]. As discussed earlier, diurnal variation in glucocorticoid levels contributes to diurnal redistribution of T cells under homeostatic conditions [21]. It is proposed that these opposing actions of glucocorticoids on the adaptive immune system are necessary to maintain immune balance [94].

#### Adrenergic signalling

Adrenergic signalling also plays a critical role in regulating the adaptive immune system. Adaptive immune cells express adrenergic receptors (primarily β2-adrenergic receptor (ADRβ2)) and signalling via this pathway regulates multiple cellular functions [100]. For example, noradrenaline suppresses T cell receptor–mediated cytokine production (IFNγ and TNFα) from effector CD8^+^ T cells [101]. Additionally, ADRβ2 signalling impacts on induction and mobilisation of Tregs [102, 103]. Twenty-four-hour variation in adrenergic signalling drives circadian variation in the adaptive immune system, the most well-established example here being diurnal variation in lymphocyte trafficking, studied in mice [22]. Adrenaline and noradrenaline are synthesised by the adrenal gland (in the adrenal medulla) for systemic release. Additionally, sympathetic neurons produce and secrete these catecholamines at discrete locations (including primary and secondary lymphoid organs [104]), providing highly localised signals [105]. Levels of circulating catecholamines reach their nadir during the night in humans [106], and the inverse in rodents [107]. Where noradrenaline rhythmicity relies on the presence of external zeitgebers such as light and food and so cannot be considered circadian, the daily oscillations in circulating adrenaline rhythms are controlled by the circadian oscillator, and mediated through the HPA axis [105, 106]. Additionally, the adrenals are innervated by neurons connected to the SCN and this pathway is directly entrained by environmental cues [105, 108].

Whilst the clock clearly influences levels of circulating adrenaline, it also impacts on local adrenergic signalling. In mice, neural inputs to ADRβ2 on lymphocytes reduce lymphocyte egress from lymph nodes by altering responsiveness of chemoattractant receptors (CCR7 and CXCR4) [109]. Daily oscillations in lymphocyte trafficking were abrogated after treatment with 6-hydroxydomapine (a neurotoxin which if administered intraperitoneally depletes peripheral adrenergic nerves and thus is used to deliver chemical sympathectomy) suggesting that adrenergic nerves were providing the signals for this diurnal regulation. Leach and Suzuki postulate that for regulation of immune function, neuron-derived adrenaline in peripheral tissues may dominate over circulating adrenaline [105].

Additional studies in human volunteers reveal a more complex picture [23]. As demonstrated in mice, CD4 + and CD8 + T cells show pronounced circadian rhythms in the blood with numbers highest during the night (02:00). However, by defining different subsets of these cells, it was revealed that CD8 + effector T cells (CD45RA + CD62L-) exhibit an inverted distribution, with elevated blood levels during the daytime [23]. Through careful manipulation of cortisol and adrenaline levels within physiological ranges, it was revealed that effector T cells are less sensitive to endogenous glucocorticoids than other T cell populations (naïve, central memory and effector memory). In contrast, in this study, only effector CD8 + T cells were sensitive to manipulations in adrenaline; lymph node homing T cell populations were insensitive. This work highlights different sensitivities of human T cell subsets to rhythms in endogenous hormones, and suggests further work is needed to fully understand the role of extrinsic hormone signalling on the 24-h variation in adaptive immune cell trafficking. Furthermore, despite the recognised daily variation in circulating levels and local action of glucocorticoids and adrenergic hormones, very little is understood about how this rhythmicity impacts on adaptive immunity beyond influencing cell trafficking. This area warrants further investigation and given the widespread use of glucocorticoids therapeutically had clear clinical implications.

### Chronotherapy

Chronic inflammatory diseases exhibit rhythmicity in their symptoms, pathology and biomarkers. There is potential to utilise this knowledge to improve both diagnosis and treatment in the form of ‘chronotherapy’.

### Chronotherapy—disease diagnosis

Studies in asthma patients demonstrate best how using knowledge of disease rhythmicity could both standardise and improve the diagnosis and treatment of chronic inflammatory conditions. Daily variation in asthma biomarkers, including exhaled biomarkers and cellular infiltrates within sputum, has been mapped. Wilkinson et al. identified a number of 24-h rhythmic exhaled volatile organic compounds (VOCs) in asthma patients (including acetone and isoprene) and noted rhythms in exhaled nitric oxide fraction (F_e_NO) which are absent in healthy volunteers. Critically, F_e_NO is utilised in diagnostic asthma algorithms; hence, time of sampling is critical to ensure stringent diagnosis [110]. Quantification of sputum eosinophils in severe asthma patients reveals higher eosinophil percentages in patients sampled during the morning clinic compared to those sampled in the afternoon clinic. Given that increased sputum eosinophil counts are an indicator for treatment escalation, time of sampling should be considered when making clinical decisions [87].

### Timed drug delivery

Chronotherapy can be described as altering the timing of administration of existing therapeutics to improve efficacy and reduce adverse effects. The best example of application of chronotherapy to treat chronic inflammatory diseases comes from RA. The Circadian Administration of Prednisone in Rheumatoid Arthritis (CAPRA) studies probe benefits of delayed release (4-h delay) prednisone in RA patients [111]. The rationale behind these studies was delivering the corticosteroid to coincide with the early morning rise in inflammatory cytokines. Trials, whereby delayed release prednisolone (or placebo) was administered alongside a disease-modifying antirheumatic drug, suggest that this approach has beneficial effects on morning joint stiffness [112] and fatigue [113].

### Targeting the clock

Given established links between the molecular clock and processes underlying adaptive immunity, there is clear potential to target the clock for therapeutic gain. The last decade has seen the development of novel ligands which act on components of the clock including REV-ERB [114], ROR [115] and CRY [116, 117]. In vitro studies have demonstrated the anti-inflammatory potential of clock targeted compounds, such as the CRY activator KL001 [55] and REV-ERB agonists [37]. Furthermore, the effectiveness of these molecules in models of autoimmune diseases has been shown. For example, the REV-ERB activators SR9009 and SR12418 have a protective effect in DSS-induced colitis [18, 41], EAE [18, 118] and CIA [119]. To date, these interventions have been limited to pre-clinical models, but evidence is mounting to support the notion that the clock is a *bona fide* target for the treatment of autoimmune disease. As might be predicted, some of the beneficial effects of REV-ERB targeting on autoimmune diseases are due to the influence of this nuclear receptor on Th17 cells. In EAE, the therapeutic effects of SR9009 are in part due to modulation of Th17 activity. SR9009 treatment of mice undergoing EAE resulted in reduced numbers of IL17A producing CD4 + T cells in the CNS and reduced disease scores [18, 118]. In contrast, the effects of SR9009 in CIA are proposed to be mediated by anti-inflammatory effects on innate immune cells (including macrophages) and joint resident synovial fibroblasts [119] and within the colon in colitis [41].

## Conclusions

The function of the adaptive immune system is regulated by the circadian clock. The clock contributes to regulation of homeostatic processes underlying maintenance of adaptive immunity (such as lymphocyte trafficking and development of Th17 cells) as well as adaptive responses to pathogenic challenge. This temporal control is an output of the combined action of cell intrinsic clocks and cell extrinsic rhythmic signals. Evidence is mounting to highlight the impact of rhythmic hormonal and sympathetic signals on driving rhythms in adaptive immunity. Given a paucity of data supporting the existence of robust cell intrinsic clockwork machinery within lymphocytes, it is plausible that extrinsic rhythmic signals play a more dominant role than intrinsic oscillators.

Inappropriate adaptive immune responses can lead to the development of autoimmune conditions such as MS, RA and UC. Studies in humans indicate that circadian disturbances (genetic or environmental) can have detrimental effects on the development of these diseases. This further highlights the importance of a robust and functional clock in maintaining immune homeostasis. Pre-clinical studies have revealed circadian regulation of inflammatory pathways driving these diseases. Studies are now beginning to elucidate the precise nature of these clock-immune interactions and highlight key rhythmic effector cells. Often, these are tissue-resident cells rather than circulating adaptive immune cells.

Looking to the future, it is critical that as a field we gain an enhanced understanding of circadian control of adaptive immunity. This knowledge has strong potential to enhance clinical practise, improving the diagnosis and treatment of chronic inflammatory disease and optimising the use of vaccines.

## Data Availability

Not applicable.
